# Fractal nature of groundwater level fluctuations affected by riparian zone vegetation water use and river stage variations

**DOI:** 10.1038/s41598-019-51657-0

**Published:** 2019-10-28

**Authors:** HongGuang Sun, Xiufen Gu, Jianting Zhu, Zhongbo Yu, Yong Zhang

**Affiliations:** 1 State Key Laboratory of Hydrology-Water Resources and Hydraulic Engineering, College of Mechanics and Materials, Hohai University, Nanjing, 210098 China; 2 Department of Civil and Architectural Engineering, University of Wyoming, 1000 E. University Ave., Laramie, WY 82071 United States; 3 State Key Laboratory of Hydrology-Water Resources and Hydraulic Engineering, Hohai University, Nanjing, 210098 China; 4 Department of Geological Sciences, University of Alabama, Tuscaloosa, AL 35487 United States

**Keywords:** Hydrology, Statistics

## Abstract

Groundwater systems affected by various factors can exhibit complex fractal behaviors, whose reliable characterization however is not straightforward. This study explores the fractal scaling behavior of the groundwater systems affected by plant water use and river stage fluctuations in the riparian zone, using multifractal detrended fluctuation analysis (MFDFA). The multifractal spectrum based on the local Hurst exponent is used to quantify the complexity of fractal nature. Results show that the water level variations at the riparian zone of the Colorado River, USA, exhibit multifractal characteristics mainly caused by the memory of time series of the water level fluctuations. The groundwater level at the monitoring well close to the river characterizes the season-dependent scaling behavior, including persistence from December to February and anti-persistence from March to November. For the site with high-density plants (Tamarisk ramosissima, which requires direct access to groundwater as its source of water), the groundwater level fluctuation becomes persistent in spring and summer, since the plants have the most significant and sustained influence on the groundwater in these seasons, which can result in stronger memory of the water level fluctuation. Results also show that the high-density plants weaken the complexity of the multifractal property of the groundwater system. In addition, the groundwater level variations at the site close to the river exhibit the most complex multifractality due to the influence of the river stage fluctuation.

## Introduction

Groundwater level fluctuations driven by various hydrological processes can evolve with spatiotemporal scales, since groundwater is a complex dynamic system with non-stationary and scale-dependent input, output, and response^1,2^. For example, groundwater input can be affected by hydraulic properties of aquifers and other hydrological processes including precipitation, runoff, infiltration, and evaporation. These properties and processes vary with the temporal and spatial scales. Consequently, the different temporal or spatial scaling features can reflect crucial hydrological features of the groundwater system.

Scaling behaviors of the groundwater system had been explored by various studies. For example, the spectral method was used to investigate the fractal scaling in the hourly records of groundwater levels for seven monitoring wells located in the Walnut Creek watershed, USA^3^. Li and Zhang^1^, Rakhshandehroo and Amiri^4^, and Zhu *et al*.^5^ employed detrended fluctuation analysis (DFA) to quantify fractal dynamics of groundwater systems. The complex scaling behavior still remains a challenge, especially when it changes with the temporal and/or spatial scales, in the prediction and quantification of subsurface processes^3,6^. Generally, the fractal structure of the groundwater level is determined by a power law exponent based on the assumption that the scaling is independent of space and time in DFA. The temporal and spatial changes in the scale structure of the groundwater level fluctuations are common (for example, there are many crossover timescales with different scale exponents), which cannot be reliably characterized by a fixed mathematical form. In this case, the DFA cannot describe detailed behaviors of fractal scales. Multifractal detrended fluctuation analysis (MFDFA) has been introduced to overcome the limitation of the DFA^7^. As an effective tool for fractal analysis, the MFDFA method has been successfully applied to analyze complex phenomena in the heart rate dynamics^8,9^, earth sciences^10^, stock market^11^, groundwater level fluctuations^4^, precancers detection^7^, the morphological differences between himalayan glacial and fluvial^12^, Mycobacterium tuberculosis genome^13^, and sunspot^14^. The local Hurst exponent is a useful tool to characterize the multifractal behavior of groundwater level fluctuations affected by the plant water use and river stage variations in space and time. Moreover, it can shed light on the prediction of the trend of groundwater level fluctuations.

To the best of our knowledge, MFDFA has not been used to distinguish the scaling characteristics of groundwater level due to plants water use and the other temporal scaling processes. Therefore, the main objective of this study is to explore the coupled effect of plant water use and other processes such as river stage variations on the fractal behaviors of groundwater level fluctuations. The rest of this work is organized as follows. Section 2 briefly introduces the study site and available water level data, including both the groundwater level time series in the riparian zone and the river stage variations. Section 3 analyzes the fractal scales of the observed groundwater level and river stage. Section 4 summarizes the main conclusions of this study. Section 5 introduces the MFDFA method and the multifractional spectrum based on the local Hurst exponent.

## Study Site and Data Collection

**Figure 1:**
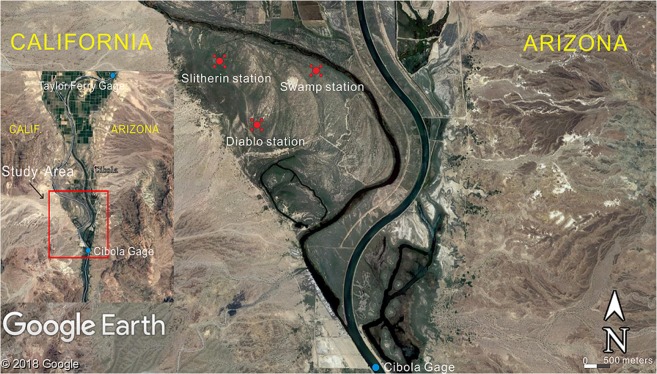
Location map of the Cibola study site showing the river stations and the groundwater monitoring wells. The map was created using the software CorelDRAW and the original pictures were obtained from Google Earth (https://www.google.com/earth/).

The site is located at the Cibola National Wildlife Refuge along the lower Colorado River, United States, with abundant observations for the groundwater levels and river stages, providing the ideal time series data set for this study^5^. Three groups of groundwater monitoring wells were drilled in the Tamarisk Ramossisima (salt cedar) stands in the study area. Tamarisk is an obligate phreatophyte that uses groundwater as its major water supply. There are 5 wells in each group, with 1 well at the tower site and 4 wells installed about 100 m on the NW, NE, SW and SE corners (Fig. 1). We selected three groundwater monitoring wells at the tower site, called Swamp, Slitherin and Diablo. They are located in 200 m, 800 m, and 1,600 m away from the Colorado River, respectively, in low-, high-, medium-density Tamarisk stands, respectively. Two gauges, known as Taylor Ferry and Cibola gauge, were used to record river stages. Taylor Ferry gauge lies upstream of the study area, and Cibola gauge is located downstream. Groundwater and river level data were collected using pressure transducers and automated data-loggers at 15-min intervals and 1-h intervals, respectively. We selected a full year record of both groundwater level and river stage data from late 2007 to late 2008 for examining the fractal nature of water level time series.

## Results

**Figure 2:**
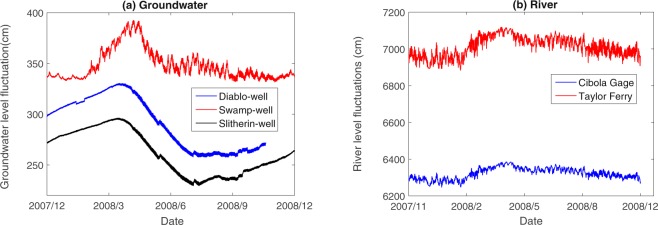
Water level fluctuations at (**a**) three monitoring wells and (**b**) two gauges (note that there was a lack of one month of record at Diablo).

The water level fluctuations depicted in Fig. 2 illustrate that the groundwater table variation observed at the two wells Diablo and Slitherin have a similar trend, which is different from the well Swamp. The changes of groundwater level at Swamp and the river stage have similar characteristics likely due to their proximity. The fluctuations of water level during spring (March, April, and May) and summer (June, July, and August) are higher than autumn and winter at wells Diablo and Slitherin. The combination of many factors contributes to the observed water level variations through the season and generates complex temporal scaling characteristics of the water levels at different locations.

**Figure 3:**
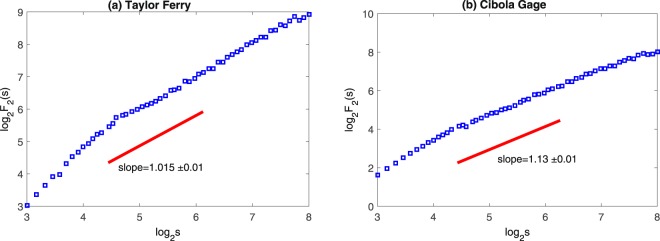
Power-law relationships between log_2_ *F*_2_(*s*) and *logs* (*s* in the units of hour) for the surface water levels at the two gauges (**a**) Taylor Ferry and (**b**) Cibola gauge. The solid lines are the best-fit results using DFA1.

**Figure 4:**
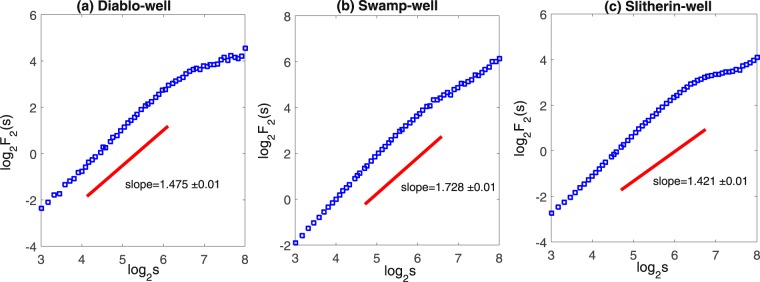
Power-law relationships between log_2_ *F*_2_(*s*) and *log s* (*s* in 15 minutes) for the water levels at the three groundwater monitoring wells (**a**) Diablo-well (**b**) Swamp-well and (**c**) Slitherin-well. The solid lines are the results fitted by DFA1.

The MFDFA performs the best when the Hurst exponent of the signal is between 0.2 and 0.8. To efficiently apply the method of MFDFA, Eke^15^ suggested obtaining an approximated value of the generalized Hurst exponent *H*_2_. When the Hurst exponent is between 0.2 and 0.8, we can directly employ MFDFA without any transformation; while when *H*_2_ is between 1.2 and 1.8, the time series should be differentiated before applying MFDFA. The generalized Hurst exponent (represented by the slope of the solid line shown in Figs 3 and 4) is calculated to determine whether the signals are the most suitable sequence to run the MFDFA. To analyze features of the original time series, the output variables such as *H*_*q*_ and *H*_*t*_ (here *H*_*q*_ is the q-order Hurst exponent, and *H*_*t*_ is the local Hurst exponent) must be adjusted because the output variables represent features of the transformation series. For example, the Hurst exponents of the groundwater level in the three monitoring wells, Diablo, Swamp, and Slitherin, are 1.475, 1.728, and 1.421, respectively. The series of groundwater levels should then be converted by one-order difference. But the time series of the two river stage fluctuations do not need to be converted before applying the MFDFA because the exponents are between 0.8 and 1.2. Table 1 ^16^ summarizes the ranges of the Hurst exponent estimated by the DFA and the corresponding conversion of output parameters, where *p*_*h*_ denotes the probability distribution of the local Hurst exponents and *D*_*h*_ represents the multifractal spectrum. It should be emphasized that the analysis and discussion presented in this paper focus on the original series although the series transformation and parameter adjustment have been used.

**Table 1:** Conversion of the time series *X* and adjustment of the related output parameters.

*H*_2_	Conversion	*H*_*t*_, *H*_*q*_	*p*_*h*_, *D*_*h*_
<0.2	cumsum (*X*-mean (*X*))	−1	0
0.2–0.8	\	0	0
0.8–1.2	\	0	0
1.2–1.8	diff(*X*)	+1	0
>1.8	diff(diff(*X*))	+2	0

The plots of log_2_ *F*_2_(*s*) versus log_2_ *s* depicted in Figs 3 and 4 show that the time series of water levels do not exhibit the monofractal scaling behavior. The plots also show the slopes of the curves of the three monitoring wells change obviously at log_2_ *s* = 6.59 (*s* ≈ 24 hrs). This change is mainly because the minimum cycle of plant water use and groundwater ET is about one day^17,18^. Besides, the ‘kink’ appeared around log_2_ *s* = 4.58 (*s* ≈ 24 hrs) for river stages since river flow is mainly constrained by the scheduled release of water coming from the Colorado River dams owning daily variation. Our analysis results presented here also verified the finding in the previous work^5^. These results further confirm that the scaling exponents can well reveal the seasonal property of the groundwater level fluctuations. However, it should be recognized that the generalized Hurst exponent cannot provide more information about the time series (e.g. multifractality). Therefore, it is necessary to further employ the MFDFA method to analyze the time series.

**Figure 5:**
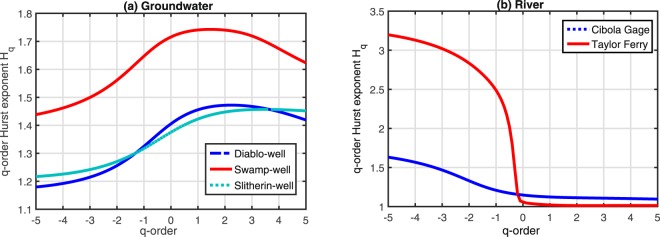
q-order Hurst exponents *H*_*q*_ vs. *q* for the time series observe at (**a**) three groundwater level monitoring wells and (**b**) two river stage gauges.

The q-order Hurst exponents *H*_*q*_ versus *q* (Fig. 5) of the time series of the groundwater level at three wells and the river level at two gauges exhibit the non-linear relation between the two variables (i.e. *H*_*q*_ strongly depends on *q*), showing that the time series have multifractal characteristics. Many interrelated factors contribute to the complex temporal scaling behaviors of water level variations, including controlled water release, groundwater ET, plant covers, interaction between the river and the riparian zone groundwater, rainfall, slope, and soil moisture conditions, among others. Moreover, when q < 0, the dramatic change of *H*_*q*_ at the Taylor Ferry site indicates the more complex multifractal feature of the river level fluctuations.

**Figure 6:**
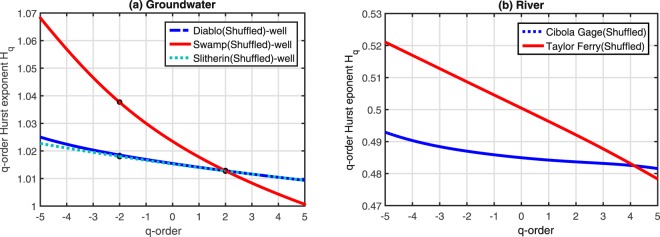
*H*_*q*_ vs. *q* relationships for the shuffled water level time series at (**a**) three groundwater monitoring wells and (**b**) two river stage gauges.

Figure 6 reveals that the q-order Hurst exponents *H*_*q*_ of the shuffled time series of water levels observed at the three wells and two gauges are changing slowly with *q*. Therefore, eliminating long-range memory of the time series seriously destroys the multifractality of the time series. It is reasonable to conclude that the multifractality of the observed time series is mainly caused by long-range temporal correlations of the time series themselves. However, the fat-tailed probability distributions of the water level variations are also an influential factor because the relationships of *H*_*q*_ versus *q* are not linear, especially at the Swamp site and Cibola gauge.

**Figure 7:**
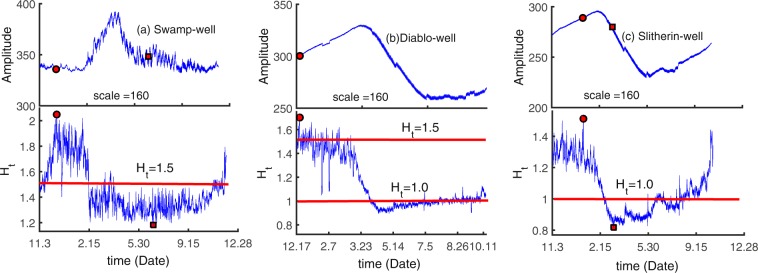
Local Hurst exponents and the original groundwater level fluctuations at the three groundwater level monitoring sites (**a**) Swamp-well (**b**) Diablo-well and (**c**) Slitherin-well. The dots and squares in the plot represent the maximum and minimum values of the local Hurst exponent, respectively.

We investigate the transition of scale structure at the study site using the local Hurst exponent. The local Hurst exponent *H*_*t*_ is a function of time, which reveals the dynamic properties of the multifractal process. The window size of 160 points (about 1.7 days at the 15-minute interval for the monitoring wells, but 6.7 days at the 1-hour interval for the two river stage gauges) is selected to obtain sufficient statistical information and describe in detail the fluctuations of the Hurst exponent at different times (Fig. 7).

When 0 < *H*_2_ < 0.5 or 1 < *H*_2_ < 1.5, the time series is anti-persistent. When *H*_2_ = 0.5, the sequence is white noise, where the past state of the sequence does not influence the future state. When *H*_2_ = 1, the time series is $\frac{1}{f}$ noise. If *H*_2_ = 1.5, the time series is Brown noise and the increment series is unrelated. When 0.5 < *H*_2_ < 1 or *H*_2_ > 1.5, the time series is persistent. Persistence means that if the water level increases (or decreases) for a period, it is likely to increase (or decrease) for a similar period. Anti-persistence usually indicates a likely decrease (or increase) in water levels after an increase (or decrease).

Because the local Hurst exponent evolution represents state change of time series, it may be a better index to characterize the memory feature. For the well Swamp, the results in Fig. 7 show that the local Hurst exponents are in the range of [1.5, 2.1] (representing a positive correlation) between December and February, but almost in the range of [1.2, 1.5] (negative correlation) for the other periods of record. In winter, less water might be released due to reducing water demand downstream, so the river stages have a weaker influence on the groundwater system. Consequently, the stronger memory of groundwater level results in the persistent time series of groundwater level in this season. At the Slitherin site, the local Hurst exponents are in the range of [1, 1.5] (anti-persistence) from September to February and the exponents are almost in the range of [0.8, 1] (persistence) from March to August. It illustrates that the local Hurst exponents can well characterize the seasonal variation at the site with high-density plants. For well Diablo, the local Hurst exponent fluctuates around 1.5 (Brown noise) from December to February. But from March to June, the local Hurst exponents decrease from 1.5 to 0.92 and then increase to 1. The results reveal that the seasonal character is weaker than the Slitherin site but the time series has a positive correlation in most of the spring. The growth of plants needs more water in summer and spring, which strengthens the memory of the groundwater fluctuation. It also shows that the local Hurst exponent can better express the scaling character of data series at different times.

**Figure 8:**
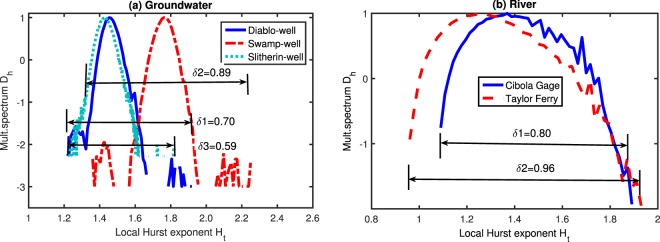
Multifractal spectrums (*D*_*h*_) estimated by the probability distribution (*p*_*h*_). The widths of spectrum of (**a**) three groundwater level time series are: *δ*_1_ = 0.70 (Diablo-well), *δ*_2_ = 0.89 (Swamp-well), and *δ*_3_ = 0.59 (Slitherin-well). The widths of (**b**) two river stage time series are: *δ*_1_ = 0.80 (Cibola gauge), and *δ*_2_ = 0.96 (Taylor Ferry).

A quantitative description of the complexity of multifractality can be obtained from the relationship between the multifractional spectrum *D*_*h*_ and the local Hurst exponent *H*_*t*_ (Fig. 8). Here the largest width is called the width *δ* of distribution, which is an indicator of the relative degree of difference between multifractal time series. Our results show that *δ* at the Swamp site is about 27% larger than the Diablo site and 51% larger than that at the Slitherin site, while the *δ* at the Taylor Ferry gauge is 20% larger than that at the Cibola gauge. These results show that the multifractality at the Swamp site is more complex compared with the Diablo and Slitherin sites. Based on this result, we may guess the width *δ* at the Slitherin site should be larger than that at the Diablo site multifractional (the groundwater level fluctuations of Slitherin and Diablo wells show a similar pattern in scaling characteristics^5^) since the Slitherin site is closer to the river. However, the width at the Diablo site is larger than the Slitherin site, which illustrates that the groundwater use of plants plays a more important role in the complexity of multifractal behavior at places away from the river.

Comparison of Figs 3 and 4 shows a similar pattern in the scaling characteristics between the river stage and groundwater level at the Swamp site. In this case, we can conclude that the multifractal characteristic of the fluctuation process of the groundwater level at the Swamp site is mainly influenced by the river stage. However, we can also conclude that water use by the high-density plants may weaken the complexity of multifractality of groundwater level fluctuations recorded with the 15-min interval in the riparian zone, compared to the multifractional spectrum width of the water level in Diablo and Slitherin wells.

For the two river stage fluctuations, the width of distribution at the Taylor Ferry gauge is larger than that at the Cibola gauge. Figure 2 shows that the river stage fluctuations at both locations have a similar frequency with a phase shift of about 9 hours due to the water travel time between these two gauges. The fluctuation at the upstream Taylor Ferry gauge has a larger amplitude and more complex multifractality.

## Conclusion

This study investigated the effect of the combination of plant water use and the nearby river stage change on temporal dynamics of groundwater in the riparian zone located at the Cibola National Wildlife Refuge along the lower Colorado River, USA. Multifractal detrended fluctuations analysis (MFDFA) was adopted to examine the multifractality of groundwater levels. Two major conclusions were drawn from this study.

First, multifractality exists in the time series of groundwater levels, which is mainly due to the memory characteristics of groundwater levels. Detailed evolution of the multifractality is sensitive to the distance between the well and the river. For well Swamp, which is close to the river (~200 m in this study), the time series exhibits a persistent scaling pattern from December to February (i.e., winter) and an anti-persistent pattern in other seasons. This is because the river stage has the weaker effect on groundwater level fluctuations in winter than that in the other seasons. For the well far away from the river (i.e., well Slitherin, which is ~800 m away from the river and represents the site of high-density plants), the groundwater level shows the anti-persistent behavior from September to February and persistence in the other months (March to August). From March to August, the high evapotranspiration rate and rapid plant growth consume more groundwater in the area of high-density vegetation, leading to a sustained decline in the groundwater level. From September to February, the diurnal signals (with the water level maxima occurring at sunrise and the minima occurring at sunset) exert the more obvious effect on the groundwater level fluctuation, enhancing anti-persistence in groundwater level evolution (i.e., the groundwater level declines after a previous increase).

Second, the width of the multifractional spectrum at the well close to the river is about 27% and 52% larger than that at wells Diablo (~1600 m, site of the medium-density plants stands) and Slitherin, respectively. Hence, we conclude that the relatively complex multifractality exists in the groundwater levels at well Swamp due to the effect of river stages on groundwater dynamics. In addition, the groundwater level dataset exhibits the weak multifractality at well Slitherin, because the high-density plants tend to alleviate the multifractal characteristics.

## Method

Multifractal detrended fluctuation analysis (MFDFA) is employed to analyze long-range correlations and multifractal property of a non-stationary time series. It can capture complex behaviors in time series and effectively exhibit their scale characteristics for the multi-scaling exponent. Since multi-scale aquifer heterogeneity and complicated controlling factors may make groundwater level fluctuations to exhibit multifractal, MFDFA is more appropriate than the standard DFA to analyze time series in this work. Its specific property was introduced by Ihlen^16^.

To illustrate this method, we consider a time series *x*(*t*)(*t* = *1, 2,* …, *N)* where *N* is the total number of measurement points with a mean value of 〈x〉. The integration in Eq. (1) first converts the times series into a cumulative sum called trajectory profile,

1 $$Y(t)=\mathop{\sum }\limits_{i\mathrm{=1}}^{t}\,[x(i)-\langle x\rangle ]\mathrm{.}$$

The trajectory profile then is divided into ${N}_{s}=int(\frac{N}{s})$ non-overlapping segments of equal length *s*. Sometimes, the integer multiple of the sub-sequence length is not exact. To effectively use the data, the remaining sequence is divided using a similar method. Therefore, 2*N*_*s*_ segments are obtained with different s values. After the sub-sequence is obtained, the variance is calculated as follows:

2 $$\{\begin{array}{rcl}{F}^{2}(k,s) & = & (\frac{1}{s})\mathop{\sum }\limits_{j\mathrm{=1}}^{s}\,{[Y[(k-\mathrm{1)}s+j]-{y}_{k}(j)]}^{2},k=\mathrm{1,}\,\cdots ,\,{N}_{s},\\ {F}^{2}(k,s) & = & (\frac{1}{s})\mathop{\sum }\limits_{j\mathrm{=1}}^{s}\,{[Y[N-(k-{N}_{s})s+j]-{y}_{k}(j)]}^{2},k={N}_{s}+1,\,\cdots ,\,2{N}_{s},\end{array}$$

where *Y*[(*k* − 1)*s* + *j*] represents the sub-sequence, and *y*_*k*_(*j*) is the solution of polynomial fitting of each segment. Linear, quadratic, cubic, and even higher order polynomials can be employed, which are called DFA1, DFA2, DFA3, …^19,20^. Then we can obtain the q-order fluctuation function by averaging the overall segments,

3 $$\{\begin{array}{rcl}{F}_{q}(s) & = & {\{\frac{1}{2{N}_{s}}\mathop{\sum }\limits_{k\mathrm{=1}}^{2{N}_{s}}{[{F}^{2}(k,s)]}^{\frac{q}{2}}\}}^{\frac{1}{q}},\,q\ne \mathrm{0,}\\ {F}_{0}(s) & = & exp\{\frac{1}{4{N}_{s}}\mathop{\sum }\limits_{k\mathrm{=1}}^{2{N}_{s}}\,\mathrm{ln}\,[{F}^{2}(k,s)]\},\,q=0.\end{array}$$

Equation (3) shows that the q-order fluctuation function *F*_*q*_(*s*) is a function of the length *s*. To reveal how the function *F*_*q*_(*s*) depends on *s* (time scale) for different *q*, we need to repeat Eqs (2) and (3) for some different time scales *s*. *F*_*q*_(*s*) also depends on the order *m* (the order of *y*_*k*_(*j*) to be fitted) of DFA (*s* ≥ *m* + 2). We can determine the scaling behavior of *F*_*q*_(*s*) by plotting *logF*_*q*_(*s*) vs. *logs* for each *q*. If the time series *x*(*t*) are long-range power-law correlated, *F*_*q*_(*s*) increases as a power law with *s*. Consequently, the q-order Hurst exponent can be expressed by Eq. (4) shown below. Then, *H*_*q*_ is called the q-order Hurst exponent. The change rate of *H*_*q*_ can be used to compare and evaluate the complexity of multifractality. A larger change rate indicates greater multifractality of the time series^21^. For the special case of *q* = 2, the MFDFA reduces to the detrended fluctuation analysis (DFA) and *H*_2_ is called the generalized Hurst exponent.

4 $${F}_{q}(s)\propto {s}^{{H}_{q}}$$

When the number of segments *N*_*s*_ in Eq. (3) becomes very small if $s > \frac{N}{4}$, *F*_*q*_(*s*) becomes statistically unreliable. Consequently, we usually exclude the case of $s > \frac{N}{4}$ in the fitting procedure when determining *H*_*q*_.

The traditional Hurst exponent analysis often provides a single value to analyze an entire time series. However, the local Hurst exponent (*H*_*t*_), a function of time, can identify the instantaneous structural changes of the time series. Illen^16^ used the local fluctuation *RMS*(*v*) to define the local Hurst exponent within a translating segment centered at each sample *v*, not within non-overlapping segments. The local Hurst exponent is obtained by Illen’s method as follows:

5 $$resRMS(v)=\,\log \,{F}_{0}(v)-\,\log (RMS(v)),$$

6 $${H}_{t}(v)=\frac{resRMS(v)}{\log \,scale}+{H}_{q}(q=\mathrm{0),}$$

7 $$\log \,scale=\,\log ({\rm{\max }}\,L)-\,\log (s),$$

8 $$RMS(v)={\{(\frac{1}{s})\mathop{\sum }\limits_{j\mathrm{=1}}^{2m}{[Y(v-m+j)-{y}_{v}(j)]}^{2}\}}^{\frac{1}{2}},$$

where ${a}_{1}=floor({\rm{\max }}(\frac{s}{2}))$, ${a}_{2}=\,{\rm{\max }}\,L-floor({\rm{\max }}(\frac{s}{2})),m=floor(\frac{s}{2})$ (floor $\frac{5}{2}=2$, for example), *s* is the window scale, *v* = *a*_1_, …, *a*_2_ and *max L* indicates the length of the *Y*(*i*), and *resRMS*(*v*) is called the residual fluctuations.

The multifractal spectrum *D*_*h*_ is merely the normalized probability distribution *p*_*h*_ of the local Hurst exponent in log coordinates. The multifractal behavior can be indicated by the relation between the multifractal spectrum (*D*_*h*_) and the local Hurst exponent (*H*_*t*_), which is an effective way to show the temporal variation of the Hurst exponent,

9 $${D}_{h}=1+\frac{\log \,{p}_{h-norm}}{\log (mean(scale))},$$

10 $${p}_{h-norm}=\frac{{p}_{h}}{{\rm{\max }}({p}_{h})},$$

where *p*_*h*−*norm*_ is obtained by normalizing the probability distribution. The procedure of the multifractal spectrum analysis was detailed in Ihlen (2012). The maximum width of the multifractal spectrum is called the spectral width and denoted *δ*. The greater spectrum width *δ* signifies the more complex multiple fractal characteristics.

Generally, there are two main causes of multifractality^22^: (1) long-range correlations of different fluctuation processes and (2) fat-tailed probability distributions of the time series. In this study, we use the method of memory destruction of time series to investigate the main reasons of water level multifractality by randomly shuffling the time series data to destroy any long-range correlations. If the shuffled time series does not show multifractality, then the multifractality of the time series is caused by long-range temporal correlations. If shuffling the time series only weakens the multifractal features of the original time series, then the multifractality is caused by these two factors simultaneously. Otherwise, the multifractality feature is caused by fat-tailed probability distributions if shuffling the time series does not change the complexity of multifractality.

## Data Availability

The data collection at the study site was supported by U.S. Bureau of Reclamation. The hourly river stages data was provided by John Weiss at the Blythe Hydrographic Office. Data requests can be addressed to corresponding author or Hongguang Sun (shg@hhu.edu.cn).
